# Modern Sociology

**Published:** 1902-08-09

**Authors:** 


### Modern Sociology.

### LACE-MAKING AS AN OCCUPATION!

There has recently been a great revival of lace as an adornment for out-of-door dresses, and ladies who have had treasures stored away, perhaps handed down to them from their grandmothers and great-grandmothers, have been unfolding the wrappings and bringing out exquisite bits of Duchesse, Honiton, and Brussels. Many of these are yellow with age and have a peculiarly musty smell, and, frequently, a stain like spilt claret, all for want of fresh air; but these defects can be remedied by careful treatment in the hands of an expert in lace, who, by dainty darns, almost invisible to the naked eye, is able to restore the delicate fabric to its original freshness and value.

The mending, cleaning, and remodelling of old lace is a special branch of art, and requires very careful training; and the mending in itself is a marvellous process to the uninitiated, such minute stitches go to the doing of it. For example, in very fine work, a small piece of muslin is laid at the back of the hole, darned into the original work, and then the superfluous muslin is cut away. This is very trying to the eyesight, but it is nothing to the strain of making some varieties of lace, notably a kind made in France a century ago. This is like a spider's web for fineness, and was actually made under conditions that in these days would make an uncommon stir among agitators for the suppression of "Home Work." This exquisite lace was made by French women in their own homes, and so fine was the thread used in its manufacture that the industry was carried on in underground cellars and by the light of a candle placed in a bottle, lest, as we are told, too great a light should break the thread. It would be interesting, and probably sad, to know how many women lost their sight in order that their wealthier sisters might go be-decked in their handicraft. Probably the wearing of it was not confined to women; for a hundred years ago men were as particular about their frills and ruffs as any fine lady could be.

Some of the oldest lace, made by darning netting, was called "opus araneum," or "spider work," but there was a coarser variety called "cut work," in which a net of threads was laid on to cloth, the cloth sewn to it in parts, and then cut away where it was not wanted; or by another method, the threads were arranged on a frame, all radiating from a common centre, and then worked into patterns. This was the old convent lace of Italy, called "Greek lace." Point laces are made with a needle on a parchment or linen pattern, the principal kinds being those of Italy, Spain and Portugal, and the modern Point d'Alençon of France.

In our English villages, a woman sitting at her door, pillow on knee and bobbins busily flying, used to be, and still is in rare parts of Bedfordshire, Buckinghamshire, and Devonshire, a familiar figure in country life. Of late, efforts have been made to revive this ancient industry, but it does not appear to offer very great attractions to the young women of to-day, though some of the lace-schools seem to flourish well. Each pillow had a neat little bag to hold the bobbins, and these latter were made in great variety. In days when there was not much book-learning, one can imagine that the bobbins formed a kind of commentary on the character of the lace-maker, and on one we have seen, made of bone, is inscribed in pin-points and sealing-wax the legend "Keep your temper, my lovely darlin'." This, presumably, was done by the admirer of the bobbin-owner, who tempered his advice with the gift of a button from his Sunday waistcoat to hang at the bobbin's tail. There is a little history even in a bobbin-head, and many of the specimens collected from English villages are weighted with square beads, moulded with sand between the finger and thumb; this points to a time previous to the invention of machines for making beads. Some of the pins, too, used in pillow-lace, have heads made of those green seed-vessels that have such an annoying habit of clinging to one's garments, when one takes a walk in the country in autumn. Perhaps the most curious bobbins, however, are those which come from Brazil; they are made of a plain strip of wood with a large nut at the end; with these, and pins which are nothing more nor less than thorns, the most exquisite lace is produced.

The quantity of lace used in our own time, both in connection with the Coronation festivities and in ordinary dress, leads to the inquiry, "Can a living be made out of lace-making at the present day?" To this question, Miss Clarke, who has recently opened a school for teaching lace-making and mending, etc., at 54 Ebury Street, S.W., replies quite definitely. A lady, she says, who has already sufficient money to live upon, can very well add to her income by making lace, provided that she is able to produce good work, and this she should be able to do after ten lessons. But in order to make a living in the ordinary sense of the term, a thorough knowledge of all the branches of cleaning, mending, and re-modelling is necessary; with this, she would have a profession which would command a living as long as lace is worn at all. The work is very suited to a lady; it is intrinsically "ladylike," and should prove attractive to those whose tastes lie in the direction of "raiment of needlework." Infinite patience is required, especially for the work of mending fine lace; this, howevar, should not deter anyone wishing to take it up. Women are always supposed to excel in detail, and perhaps those who attribute the modern woman's capacity for detailed work in other directions to their grandmothers' toil at tambour-frame or tapestry, are not far wrong.

Although it is as an occupation for ladies that we are considering the question, a passing allusion must be made to an interesting class for lace-making which is being held at the Cripples' Home and Industrial School for Girls, in Marylebone Road; this is conducted by Miss Reynolds, Miss Clarke's head teacher. It is very encouraging to those concerned in the welfare of cripples to know that great success is attending this experiment, and that some of the pupils have learnt as much in five lessons as many ladies would in twice the number. Their pillows are a new delight to these women and girls whose chief occupation hitherto has been the plaiting of straw for workhouse bonnets, and it is anticipated that a new industry may open for them. The kinds taught are Honiton, Buckinghamshire, Torchon, and Bruges.

It has been suggested that possibly even blind women might learn to make lace, and indeed when one realises how the difficulties of type-writing and mat-weaving have been overcome by the blind, it does not seem at all improbable that by first learning the pricked patterns with the fingers, the intricate movements of the bobbins and pins might be mastered. This process has to be gone through in mat-weaving, and some apparently difficult patterns are successfully carried out in the higher branches of this industry. If lace-making were found to provide a new opening for blind women, it would be an unqualified blessing for those whose means of livelihood are at present so extremely limited.

To ladies who are interested in the question of lace-making as an occupation, a visit to "Ye Olde Pillow Lace School," 54 Ebury Street, may be confidently recommended. Here they will see not only specimens of dainty laces, old and new, but an interesting collection of pins and bobbins, which are in themselves fascinating.

